# Challenges in Managing a Case of Neonatal Listeriosis in Portugal

**DOI:** 10.7759/cureus.38405

**Published:** 2023-05-01

**Authors:** Diana Simão Raimundo, Eulália Viveiros, Isabel Monteiro, Fernanda Gomes

**Affiliations:** 1 Pediatrics Department, Hospital do Divino Espírito Santo de Ponta Delgada, Ponta Delgada, PRT; 2 Neonatal Unit, Pediatrics Department, Hospital do Divino Espírito Santo de Ponta Delgada, Ponta Delgada, PRT

**Keywords:** congenital infection, maternal-fetal transmission, congenital pneumonia, listeria monocytogenes, neonatal listeriosis

## Abstract

A male neonate was born at 34 weeks due to spontaneous labor with associated fetal distress and meconium-stained amniotic fluid. The neonate presented with septic shock and congenital pneumonia shortly after birth and later neurological symptoms. *Listeria monocytogenes* was identified in blood samples, but with negative urine and cerebrospinal fluid cultures. The neonate required assisted ventilation for a period of 10 days and received high-dose and long-term antibiotic therapy. Despite the fact that the mother denied an infectious risk for listeriosis infection, she developed mild respiratory symptoms. Her microbiological investigation was negative, although it did not include placental samples. Vertical transmission in this case was presumed but not confirmed. The newborn was discharged asymptomatic at day 26 of life and has presented normal developmental evolution until present, at eight months old. *Listeria monocytogenes* is a classic but relatively rare cause of neonatal sepsis and meningitis. This case describes a clinically successfully managed case with no possible epidemiological link and illustrates the challenges in managing cases of a public health disease. In neonatal listeriosis, communication between Neonatology and Obstetrics departments, as well as with public health, is vital, and long-term follow-up is crucial to identify possible neurological sequelae.

## Introduction

We describe the case of a male preterm newborn who presented with fetal distress, septic shock, congenital pneumonia, and subsequent neurological involvement. Neonatal listeriosis with presumed vertical transmission was diagnosed.

Neonatal listeriosis is caused by *Listeria monocytogenes* and is a disease both in the scope of medical care and public health [1]. Infection may occur as sporadic cases or in the context of outbreaks, as neonatal listeriosis is an important foodborne maternal-neonatal infection [2]. It may be acquired by pregnant women through unpasteurized dairy products, meat-derived products, raw seafood, vegetables, and ready-to-eat food [2,3].

Early manifestations of neonatal listeriosis occur in the first seven days of life, mostly due to vertical transmission: transplacental due to intrauterine infection, ascending colonization of the vagina, or passing through the birth canal [4,5]. It usually manifests as sepsis, but pneumonia or respiratory distress syndrome, fever, neurological symptoms, jaundice, skin lesions, and conjunctivitis may occur [4]. Late infection is mostly diagnosed between days 7 and 90 of life and occurs due to vertical or nosocomial transmission, usually as meningitis [1,5,6]. Mortality rate is higher in early infection (20-60%) [5-7].

In Portugal, listeriosis has been a mandatory reporting disease since 2014 [8,9]. However, there is no active surveillance programme for the disease.

## Case presentation

**Figure 1:**
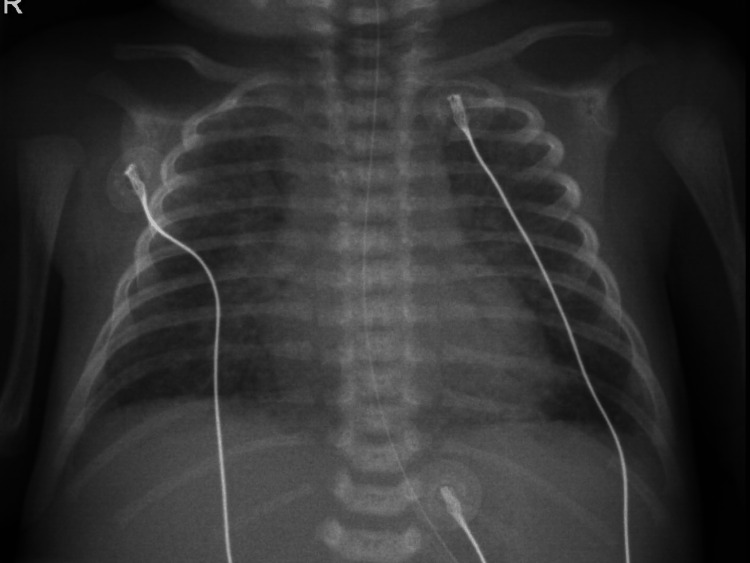
Congenital pneumonia. Chest radiograph, anteroposterior incidence, showing bilateral and diffuse interstitial pulmonary infiltrates, with air bronchogram, and partially sparing the left lung base, with no signs of pleural effusion or pneumothorax.

A male preterm infant was born by emergent cesarean section at 34 weeks of gestational age due to spontaneous labor with fetal distress. The pregnancy was otherwise uneventful. No prepartal antibiotics were given as routine screening for group B *Streptococcus* on a vaginal/rectal swab was negative. A thirty-four-year-old nulliparous woman with irrelevant past medical history presented negative screening for syphilis, hepatitis B, human immunodeficiency virus (HIV) 1 and HIV 2, and presented immunity against *Toxoplasma* and *Rubella* infections. The ultrasounds performed in the first and second trimesters were unremarkable. A third trimester ultrasound was not performed. During birth, meconium-stained amniotic fluid was noted. Neonate was born hypotonic and bradycardic, whereby tracheal suctioning and positive pressure ventilation were performed. Apgar score was 2/7/9 in the first, fifth, and 10th minutes. The infant stayed under clinical surveillance in the recovery room. At two hours of life, the newborn developed septic shock with severe hypoxemia, bradypnea, and hypoglycemia, and he was admitted to the neonatal intensive care unit (NICU). Chest radiograph revealed congenital pneumonia (Figure 1). Neonate was placed on invasive ventilation and inotropic support, and empiric ampicillin and gentamicin were started. *Listeria monocytogenes *was identified in a blood sample by the FilmArray Meningitis/Encephalitis panel (BioFire Diagnostics, Salt Lake City, Utah, United States) blood culture identification panel and confirmed by two sets of blood culture. No microbiologic agents were identified in cerebrospinal fluid (CSF) and urine cultures.

While ventilated, the neonate developed spastic movements with no hypotonia, tremor, tonic-clonic seizures, or bulging anterior fontanelle. Despite CSF was sterile, a head computed tomography (CT) scan was performed to exclude acute ischemic lesions, hemorrhage, or intraparenchymal expansive lesions that would cause the neurological picture, and no pathological findings were reported. On day 6 of life, the clinical status of the newborn deteriorated as he presented with fever, purulent conjunctivitis, and diffuse desquamative rash, as well as an increase in C-reactive protein (CRP) from 15 to 18 mg/dL. *Granulomatosis infantisepticum* was hypothesized, but it was clinically excluded since the skin changes were not typical and there was no suspicion or evidence of granulomas in other organs. Due to the presence of a central venous catheter (CVC), nosocomial staphylococcal infection was the most likely hypothesis placed; therefore, CVC was removed and IV vancomycin and ophthalmic gentamicin were added to the prescription chart. The cultures of CVC tip and conjunctival exudate were sterile. Nevertheless, improvement in general status was seen thereafter. On day 10, the neonate was extubated to noninvasive ventilation. In total, triple intravenous antibiotic therapy was performed, which included ampicillin at a dose of 300 mg/kg/day every eight hours for 21 days, gentamicin at a dose of 5 mg/kg/day every 24 hours for 14 days, and vancomycin at a dose of 30 mg/kg/day every 12 hours for 10 days. The newborn was discharged on day 26 of life, asymptomatic and in good general condition.

This case was notified to the national epidemiological surveillance system, and local public health commission was warned. Mother denied past medical history for ingestion of unpasteurized milk or cheese, undercooked meat or fish, and poorly washed raw sprouts. The mother was asymptomatic until day 2 after birth, after which she developed a mild cough and fever. Chest radiograph was unremarkable, and blood cultures were negative, but no FilmArray Meningitis/Encephalitis panel (BioFire Diagnostics, Salt Lake City, Utah, United States) was performed. The mother was given IV ampicillin and gentamicin for nine days with a resumption of symptoms. Epidemiological inquiry was performed. No associated listeriosis outbreak was identified.

At present, the infant maintains follow-up in neonatology consultation and physiotherapy sessions since discharge. At the age of eight months, the child presents adequate psychomotor development with no apparent sequelae.

## Discussion

We present a case of early neonatal listeriosis with presumed vertical transmission. A spontaneous premature labor occurred from an uneventful pregnancy. Although not specific, prematurity and meconium-stained amniotic fluid should always remind clinicians of the possibility of neonatal listeriosis [10].

The transmission of *L. monocytogenes* occurs through ingestion of contaminated food, mainly raw beef, pork, or poultry; crustaceans, shellfish, or molluscs; unpasteurized milk and derivatives; and raw or poorly washed fruits and vegetables [8]. Listeriosis infections are more likely to occur in the third trimester of pregnancy, and symptoms in pregnant women are mostly flu-like, such as fever and fatigue [11].

This infectious disease is considered a classic but relatively rare cause of neonatal sepsis and meningitis, as it occurs in approximately 8:100,000 live births [1,5,12]. Incidence of general listeriosis in Portugal in a non-global national study was estimated as 2.3:100,000 people in 2007 [13]. There are no available current data on neonatal listeriosis incidence in this country. However, the low incidence of this disease may be underestimated due to several factors. One is the occurrence of spontaneous abortions and stillbirths with unidentified causes [6]. Another factor is the relative difficulty in identifying the pathogen due to its intrinsic features. Listeria *monocytogenes* is a gram-positive bacillus with an intracellular lifecycle, one of the few invasive pathogenic organisms in neonates with such characteristic but it can easily be mistaken for diplococcus [1,12]. Correct communication with the laboratory about diagnostic hypotheses must be established. Furthermore, biological samples may not contain* L. monocytogenes* in enough quantities to be visible on direct Gram-stain [5]. For that reason, it is highly recommended to secure sufficient quantity of samples and to obtain relevant specimens such as amniotic fluid, meconium, CSF or skin lesions [1,12,14]. The lack of a rapid confirmatory test, such as Polymerase Chain Reaction (PCR) test, also contribute to the delayed diagnosis of listeriosis [5]. For all that, when *L. monocytogenes* is suspected or when there is no apparent cause to early neonatal sepsis, a good communication between clinical and nursing staff is vital to ensure quality biological samples. In the presented case, chest radiograph performed short after birth revealed bilateral and diffuse interstitial pulmonary infiltrates with air bronchogram suggestive of congenital pneumonia. Radiological findings in pneumonia from *Listeria* are similar to those seen in other bacteria pneumonias, not being useful to establish the diagnosis [1].

It is essential to have a pre-established cooperation between the Neonatology and Obstetrics services in an effort to protocol standard measures of action in cases of spontaneous premature labor, namely obtaining placental samples, since it will not be possible to obtain these samples later. In order to diagnose neonatal listeriosis from maternal-fetal transmission, placental cultures should be performed. Alternatively, maternal blood cultures and CSF culture could be used although they have lower sensitivity [5]. In the case presented, neither placental nor amniotic fluid was obtained, despite spontaneous prematurity and the presence of meconium-stained amniotic fluid. Two maternal blood cultures were performed but no FilmArray®, which could be an additional way of identifying the microbiological agent. Vertical transmission in this case was presumed but not confirmed. Neonatology and Obstetrics departments should carry out joint investigation for a more accurate diagnosis as well as better treatment and outcomes of both mother and newborn.

Skin lesions classically referred to as *granulomatosis infantisepticum *are uncommon findings in neonatal listeriosis, consisting of 1-2 mm pale spots on an erythematous base, mostly on the trunk [1]. It may be associated with granulomatous pneumonia and granulomas in the liver and other organs [4]. In the presented case, the neonate developed a diffuse desquamative rash along with purulent conjunctivitis and fever. Although it is tempting to assign all parts of a clinical picture to a single entity, especially when it is rare, it is important not to forget what is most frequent.

Regarding therapeutic options, ampicillin is the preferred antibiotic for treating *L. monocytogenes* infections, as there are virtually no resistances. To achieve effective local levels of antimicrobial concentrations, ampicillin must be given near the higher recommended range [1,7,15]. In this case report, a high dose of IV ampicillin 300 mg/kg/day every eight hours was performed. An aminoglycoside, such as gentamicin, can be added for potential synergistic benefit, but it should not be administered alone as it has very poor penetration into intracellular spaces [1,15]. The recommendation on duration of therapy to neonatal listeriosis is not well established but mostly varies from 10 days to four weeks [16].

As to *L. monocytogenes* meningitis, there are no studies assessing the optimal duration, but guidelines recommend 21 days of therapeutic or longer [17]. In the presented case, albeit CSF and head imaging were normal, ampicillin was extended to 21 days due to the presence of neurological symptoms. No neurological sequelae have been found so far. About 10% of infected newborns may develop long-term neurological sequelae [1]. In that regard, long-term follow-up during childhood is crucial.

## Conclusions

This case report describes a successful clinical management of a newborn with neonatal listeriosis but failing to clearly identify maternal-fetal transmission. Neonatal listeriosis is not an isolated clinical disease; it is simultaneously a disease of the mother and the neonate as well as a public health issue within a possible outbreak. The learning points we intend to share with this case are (1) to obtain a sufficient quantity of samples and to obtain relevant newborn (blood, CSF, amniotic fluid, or skin lesions) and maternal specimens (placenta, blood, CSF) in order to maximize the chances of finding the responsible agent; (2) to communicate within Neonatology department (doctors and nurses) and with Obstetrics department and the clinical laboratory.
